# Design and Properties Analysis of Novel Modified 1-3 Piezoelectric Composite

**DOI:** 10.3390/ma14071749

**Published:** 2021-04-02

**Authors:** Jiacheng Wang, Chao Zhong, Shaohua Hao, Likun Wang

**Affiliations:** 1 Beijing Key Laboratory for Sensor, Beijing Information Science & Technology University, Beijing 100101, China; 2019020178@bistu.edu.cn (J.W.); 20192289@bistu.edu.cn (C.Z.); 2 School of Electronic Engineering, Beijing University of Posts and Telecommunications, Beijing 100876, China; haoshaohua@bupt.edu.cn

**Keywords:** piezoelectric composite, structure, properties, decoupling

## Abstract

With the increasing demand for energy exchangers in underwater acoustic equipment, a modified 1-3 piezoelectric composite material is fabricated based on three-component phases. The new material outperforms the traditional two-phase 1-3 structure. Flexible silicone rubber polymer strengthened the piezoelectric composite and the properties of modified 1-3 piezoelectric composite have been tested by method of finite element simulation and experiment, respectively. This modified material has a high electromechanical coupling coefficient; the maximum can reach 0.684 and −3 dB bandwidth is superior to the two-phase 1-3 type. At the same time, the modified phase 1-3 type structure has an excellent decoupling effect. Silicone rubber can reduce the negative coupling vibration of epoxy resin, the vibration model simplification of piezoelectric composite, and the result of the experiment and simulation has good consistency.

## 1. Introduction

With the increasingly high requirement for information and intelligent technology, a great breakthrough has been made in material science, which is intelligent materials. The application of such materials has contributed to the development of modern intelligent instruments [1,2]. As functional materials applied in hydroacoustic sensing and image detection, 1-3 piezoelectric composites have become increasingly mature and play a central role in the sensitive elements of transducers [3]. Compared with conventional piezoelectric ceramic materials, they have better physical properties and development qualities, thereby attracting wide attention from scholars [4,5,6].

The early applications of piezoelectric materials were mostly in single crystals. In addition to quartz, scientists were extremely enthusiastic about researching Rochelle salts. The ferroelectricity of such salts was first discovered in 1921 by J. Valasek [7] through a dielectric anomaly test. Compared with conventional quartz crystals, the piezoelectric coefficient of Rochelle salts can be up to 1000 times higher [8]. However, certain disadvantages are identified during the application of this material. Rochelle salt has poor mechanical properties and low operating frequency. Thus, it has been gradually replaced with other piezoelectric materials in some fields [9,10]. After the Second World War, scientists discovered a series of piezoelectric materials with different properties, such as ammonium dihydrogen phosphate, lithium niobate, and other piezoelectric single crystals [8]. However, they are not currently used on a large scale due to their partial defects in mechanical strength, piezoelectric properties, and stability [11,12,13].

In the 1950s, following piezoelectric single crystals, piezoelectric ceramics were developed by scholars, such as S. Robert and Jaffe [14,15]. Their research enabled piezoelectric materials to have a broad application prospect. Traditional piezoelectric ceramics are mainly titanates and lead-containing compounds [16], where the lead zirconate titanate series (PZT ceramics) remains widely used today because of their Curie temperature, piezoelectric constants, and electromechanical coupling coefficients being comparably high. However, using piezoelectric ceramics alone as sensitive elements leads to poor matching with water and biological tissues due to their high acoustic impedance. The high density and brittleness of piezoelectric ceramics indicate that the material is prone to breakage and fracture from mechanical vibrations [17]. Therefore, they are unsuitable for large area use in transducer arrays; their small hydrostatic piezoelectric constants limit the sensitivity of hydrophones [18]. The piezoelectric ceramic block has large lateral coupling [18], and the thickness vibration mode is impure, thereby limiting the application of hydroacoustic transducers in medical devices. Therefore, an inevitable trend in the development of piezoelectric materials is to design new materials with low acoustic impedance, high electromechanical coupling coefficient, small density, and high hydrostatic piezoelectric constants while retaining the advantages of traditional piezoelectric ceramics [19,20,21,22,23,24]. These conditions require the use of piezoelectric composites. Such composites are piezoelectric materials made of piezoelectric ceramics and polymers in a certain connectivity and addition ratio [25]. In 1972, Japanese scholars developed PVDF-BaTiO_3_ material, thereby beginning a new era of research on piezoelectric composites. [26] The focus of research is no longer limited to single-crystal and single-phase piezoelectric ceramics. R.E. Newnham et al. [27] proposed a series-parallel complex model based on the structure of piezoelectric composites. This model enlightened the development of 10 connected structures of piezoelectric composites [7,28], namely, 0-0, 0-1, 0-2, 0-3, 1-1, 1-2, 1-3, 2-2, 2-3, and 3-3. Among them, the first number represents the connected dimension of the piezoelectric phase, and the second number represents the connected dimension of the polymer phase. Piezoelectric composites possess the advantages of piezoelectric and polymer phases. Thus, they are the focus of the current research field.

Compared with other types of piezoelectric composites, type 1-3 piezoelectric composites have better hydroacoustic properties [29] and are easier to prepare. Thus, they have been widely studied and applied. The conventional 1-3 type is a two-phase piezoelectric composite composed of 1D linearly arranged piezoelectric pillars and 3D connected polymers. This composite retains the original advantages of traditional ceramic materials and has better performance. It has the following characteristics: (1) low characteristic impedance (approximately 20 MRayl) [30], which reduces the overall acoustic impedance of the composite (e.g., the impedance for 618 epoxy resin is approximately 3 MRayl). The acoustic impedance of the overall material gradually decreases with the increase in the volume ratio of the polymer phase, making the material easy to acoustically match with water or biological tissue. (2) The electromechanical coupling coefficient of thickness is large [29,31]. This condition is because the polymer separates each piezoelectric small column so that the transverse vibration mode of the composite is suppressed. (3) Low dielectric constant [29,32]. In 1-3 piezoelectric composites, the relative permittivity of piezoelectric ceramics incorporated with polymer phase is remarkably lower than that of ceramic monomers because the dielectric constant of the polymer phase is smaller than that of the piezoelectric phase. The static capacitance of piezoelectric composites with lower dielectric constants is relatively small. Thus, the hydroacoustic transducer prepared with 1-3 piezoelectric composites has a great reception sensitivity.

Currently, 1-3 piezoelectric composites are mainly prepared by using a dice-filled method. This method is commonly used, with an extremely short preparation cycle and a relatively simple process. The preparation steps are as follows [33,34]: first, the polarized piezoelectric ceramics are cut along the parallel and perpendicular directions to make a ceramic skeleton. The sample ceramic skeleton is then cleaned and dried with acetone and other chemical reagents. A certain amount of polymer is poured into the skeleton gaps and cured by vacuum; finally, the preliminary finished product is polished, and electrodes are plated on the upper and lower surfaces. Some scholars often used arrangement-casting method to prepare 1-3 type composites in the early days [35,36,37]. The basic idea is to arrange a certain number of circular piezoelectric rods in a mold, then inject the polymer for curing, and finally demold to prepare the electrodes. This method is not used nowadays due to its complex process. C. Negreira et al. [35] explored the nonperiodic nature of the material using the arrangement-casting method and improved the elasticity of type 1-3 piezoelectric composites. K. A. Klicker et al. [36] derived the theory of type 1-3 piezoelectric composites on the basis of series-parallel theory and prepared this composite by using the arrangement-filling method. Its piezoelectric constant *d*_h_ is three times higher than that of PZT-501A, and the hydrostatic piezoelectric constant *g*_h_ is increased by 25 times. The parameters of the 1-3 piezoelectric composites such as electromechanical coupling coefficient and acoustic impedance, are not affected by the shape of the PZT rods, but by the volume fraction of the ceramic phase in the vibrator and the effective cross-sectional area of the PZT rods. However, the preparation cycle of the arrangement-filling method is extremely long. PZT powder is prepared by using traditional methods, such as extrusion and molding for circular piezoelectric rods, which have a low fault tolerance and are difficult to prepare. The 1-3 piezoelectric composite materials are prepared by using various methods, such as the injection mold forming method and laser cutting method [29,38]. However, these methods are no longer used due to their complex processes and expensive equipment.

In recent years, many researchers have enhanced the performance of conventional 1-3 piezoelectric composites by changing the structure or constituent phases. Wang et al. [39] prepared a lead-free piezoelectric material containing barium tin titanate with a piezoelectric constant *d*_33_ up to 1100 pC/N. Habib et al. [40] prepared a lead-free titanate-based piezoelectric ceramic by using the solid-phase reaction method (BF30BT-100xlf) with an inverse piezoelectric coefficient *d*_33_* up to 500 pm/V. Ke et al. [41] prepared 1-3 piezoelectric composites using niobate (KNN) and epoxy resin with electromechanical coupling coefficients up to 0.7. Li et al. [42] made 1-3-2 piezoelectric type piezoelectric composites using ferroelectric single crystals while preserving the connectivity of the original materials. Their electromechanical coupling coefficient can reach up to 0.75. In practical applications, ferroelectric single crystals are expensive and have large characteristic impedance, thereby greatly limiting them in massive production. He [43] et al. prepared air-based 1-3 piezoelectric composites with a 0.4 ceramic (PZT-5H) ratio using 3D printing technology and experimentally verified that the air-coupled transducer prepared with this material has a low signal-to-noise ratio and an electromechanical coupling coefficient of up to 0.7. However, the injection molding method is incorporated during the preparation, indicating that it has a long preparation cycle. Rouffaud et al. [44] changed the spatial arrangement of the piezoelectric phases to make a supercell type 1-3 piezoelectric composite. This method reduces the spurious phenomena of the conventional cut-and-pour method and significantly improves the electromechanical properties.

Nowadays, 1-3 piezoelectric composites and their derivative class designs are mostly prepared from piezoelectric ceramics and rigid epoxy resins, and the piezoelectric oscillators of hydroacoustic devices made of such material are stiff and less bendable. This condition indicates that these composites are difficult to form into the shape needed for the curved shells of devices, such as underwater unmanned vehicles and high-frequency sonar [45]. Curved transducers can improve certain beam opening angles and operating bandwidths compared with planar ones. These transducers are the most promising design at present. However, the existing curved 1-3 piezoelectric composites have long preparation cycles and low tolerance [35,46]. Therefore, the design of an easy-to-prepare and flexible 1-3 piezoelectric composite is the key to addressing the existing problems.

A modified 1-3 piezoelectric composite material is designed on the basis of three component phases in this paper to improve the electromechanical coupling coefficient of thickness, working bandwidth, and reduce the characteristic impedance of materials. The novel materials are fabricated by dice-filled technology. The experimental results show that the modified 1-3 type three-phase piezoelectric composites have higher electromechanical coupling coefficient and working bandwidth, lower characteristic impedance, and better decoupling effect than the traditional two-phase 1-3 piezoelectric composites. These modified composites have a great application prospect in nondestructive testing and medical ultrasound [47,48].

## 2. Materials and Methods

### 2.1. Design of Modified 1-3 Piezoelectric Composite Structure

**Figure 1:**
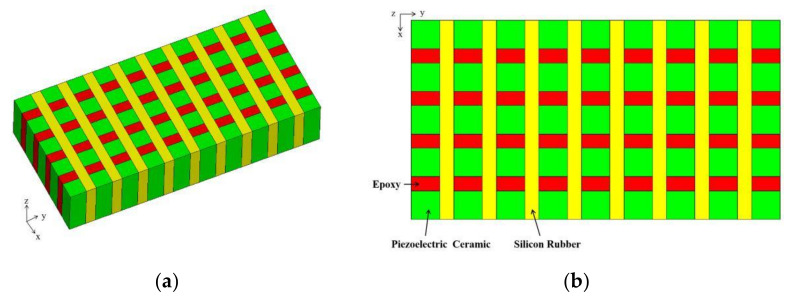
3D structure diagram (**a**) and 2D diagram (**b**) of modified 1-3 piezoelectric composite material.

Figure 1 shows the structures of modified 1-3 type piezoelectric composite. This composite is based on three constituent phases, where the green part is the 1D piezoelectric phase, the red part is the epoxy resin, and the yellow part is the flexible silicone rubber phase. Epoxy resin has a large Young’s modulus, thereby supporting the piezoelectric composite and improving the vibration stability. However, excessive amounts of epoxy resin can cause negative coupling vibration of the material. Negative vibration coupling, which is a vibration in the direction of nonthickness, occurs when the rigid polymer involved vibrates in piezoelectric composites. In the case of 1-3 type piezoelectric composite material, epoxy resin is stiff after solidification, and its width increases with the increase in the amount of epoxy resin. Every piezoelectric rod drives the epoxy resin to vibrate around, causing strong polymer vibration energy. Thus, the whole material’s vibration leads to the nonthickness direction. Therefore, the admittance curves of materials have multiple coupling resonance peaks. The energy of the composite thickness vibration is lost. Silicone rubber, a type of flexible polymer, promotes the piezoelectric vibration of each pillar and improves the ability of the material itself during electromechanical conversion. Compared with epoxy resin, silicon rubber usually has an extremely small Young’s modulus. Thus, it has higher flexibility. When the piezoelectric phase vibrates, the vibration of each piezoelectric rod is not limited. However, when the composite material is used for extremely long periods, the silicone rubber exhibits aging and deformation. Therefore, excessive silicone rubber causes the electrode on the material surface to fall off, resulting in the deformation of the whole material [49]. Given these factors, the modified 1-3 piezoelectric composite is designed to avoid the potential negative effects of the two types of polymers.

The structural arrangement of the modified piezoelectric composites is the same as that of 1-3 type, which belongs to the structure of 1-3 type piezoelectric composite derived class. The attenuation coefficients of the two types of polymer phase are large. Thus, they can simultaneously attenuate acoustic radiation in the x and y directions, thereby enhancing the thickness of the piezoelectric composite vibration. The vibration energy of the material is concentrated in the single z-direction. At the same time, as a rigid polymer phase, epoxy resin can apply a mechanical clamping effect in the x direction and promote the vibrator’s stability. As a flexible polymer phase, silicone rubber can bend the material moderately along the y direction, thereby enabling the material to form a curved structure and to easily bend toward a single direction. Such piezoelectric composite has not been reported previously.

### 2.2. Finite Element Simulation and Model Analysis

The practicability of piezoelectric composite material should be considered first when designing such materials. Thus, we conducted finite element analysis (FEA) on ANSYS 15.0 (ANSYS, Inc., Pittsburgh, PA, USA) software on the structure of piezoelectric composite materials before the test. In the finite element analysis, models are usually divided into multiple small units, and the final result is calculated by summation. When the mesh is divided in accordance with the model, the smaller the mesh, the more accurate the calculation. In this simulation, the resonant frequency of the material is approximately 450 kHz, and the acoustic wavelength in the air is approximately 7.5 mm. For numerical accuracy, a wavelength is usually divided into no less than 10 segments, that is, the mesh size is up to 0.75 mm. To obtain better experimental data, we set the mesh to 0.2 mm, and the mesh type as the hexahedral element.

**Figure 2:**
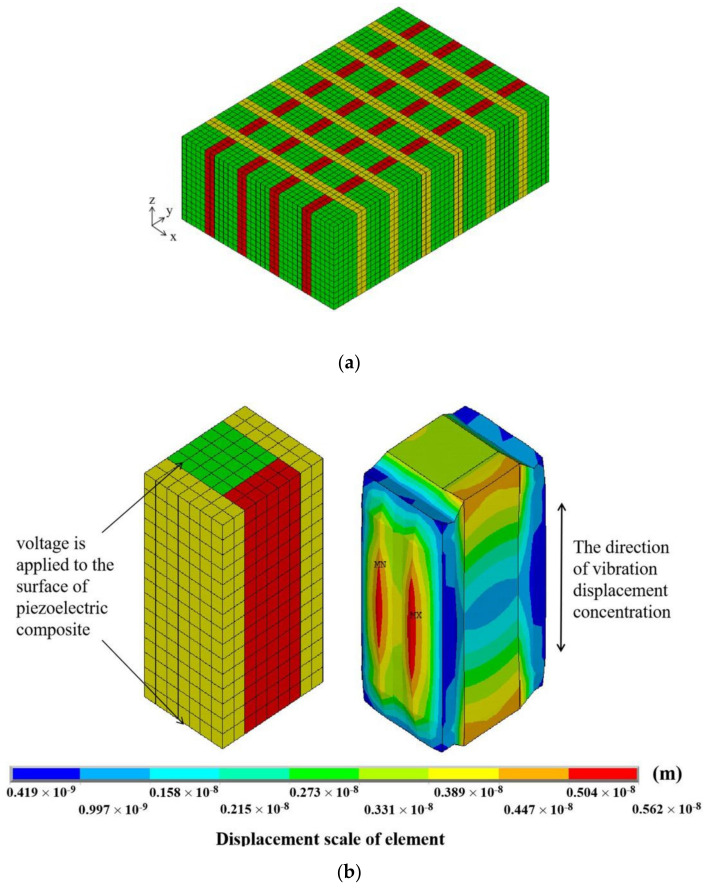
Mesh division of finite element analysis (**a**) and its element vibration displacement (**b**).

The modified 1-3 piezoelectric composite material after the division of finite element mesh is shown in Figure 2, where the green part is the piezoelectric phase PZT-5A, the red part is 618 epoxy resin, and the yellow part is 704 silicone rubber. In the preprocessor, the length, width, and thickness of the piezoelectric vibrator are set to 63, 29.5, and 3 mm, respectively. The polarized piezoelectric ceramics are anisotropic, and the polymer phases are isotropic. Thus, the parameters can be set in accordance with Table 1. The finite element mesh size of the material is set to 0.2 mm, and the automatic “Sweep” function is used to divide the finite element mesh. We obtain 696,938 elements using this method. A simple harmonic AC voltage of 1 V is applied between the upper and lower surfaces of the material as an external excitation signal. The electrode covered by the vibrator is extremely thin. The effect of its thickness is negligible in the simulation analysis. The displacement boundary conditions are set on each side of the material (XOZ plane). Air damping [13,28] refers to the physical quantity that reduces the resonance amplitude when a vibration system is subjected to external air resistance that causes the material to vibrate. For calculation accuracy, the influence of air on the working vibrator is considered. Here, the air damping coefficient is set to 0.02. The frequency range is set from 350 to 700 kHz in harmonic response analysis. The vibration of the composite material in the air is simulated. The model and vibration after being divided are shown in Figure 2.

Piezoelectric composites usually have three vibration modes. The first is the transverse vibration mode. In this mode, the polarization direction of the material is parallel to the electric field’s direction; the wavelength of the sound wave is similar to the length of the material. When an AC voltage signal is excited, the material vibrates along its length. The second is the shear vibration mode. In this mode, the polarization direction of the material is perpendicular to the direction of the electric field. This condition causes the strain of the material to be generated on the geometric shear surface of the material. The last one is the thickness vibration mode. As shown in Figure 2, the polarization direction of the material is oriented vertically upward along the z-axis, and the electrodes cover the upper and lower surfaces of the material. Therefore, the electric field direction is parallel to the polarization direction. It is larger than the wavelength of the sound wave. Thus, the vibration energy of the modified 1-3 piezoelectric composite material is concentrated in the thickness direction.

**Figure 3:**
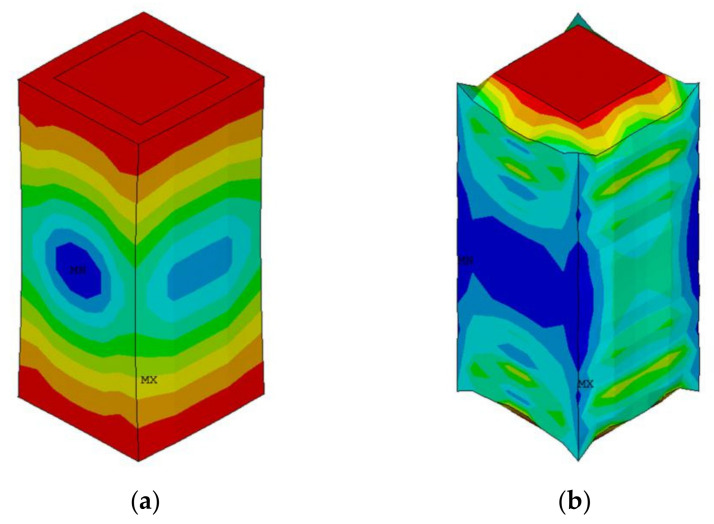
Displacement deformation diagram of each pure epoxy resin (**a**) and silicone rubber (**b**) composite element.

The positive effects of the thickness vibration modes of the two polymers in the composites on the vibrator are different. The different effects of pure epoxy and pure silicone rubber on piezoelectric rod are introduced by taking two types of 1-3 piezoelectric composite elements as examples. In Figure 3, the influences of the two polymers on 1-3 piezoelectric composites are described. The displacement of pure epoxy element is 0.388 × 10^−8^ m, while that of pure silicone rubber element is 0.422 × 10^−8^ m. The inside of the material is assumed to be uniform; a unit of pure epoxy and pure silicone rubber 1-3 type composite are adopted in the ANSYS simulation to explore the effect of the two polymers. As shown in the displacement diagram, the rigid epoxy resin inhibits the vibration of the piezoelectric column and limits its vibration displacement. However, the epoxy resin acts as a mechanical clamp to effectively suppress the lateral vibration of the material. The silicone rubber is relatively soft, and the piezoelectric column wrapped by silicone rubber has a large displacement, promoting column vibration. In the modified 1-3 novel type, epoxy resin has high Young’s modulus and stiffness. This condition effectively suppresses the transverse vibration of the vibrator and plays a mechanical clamping role in the X direction of the vibrator. By contrast, silicone rubber, as a flexible polymer, has small Young’s modulus and can effectively promote the thickness vibration of each piezoelectric column.

### 2.3. Fabrication and Experiment of Modified 1-3 Piezoelectric Composite

**Figure 4:**
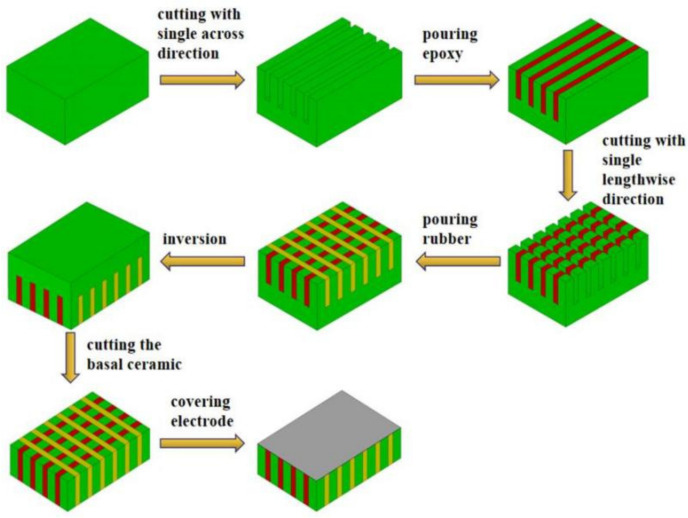
Flow-chart of fabrication of modified 1-3 piezoelectric composite.

Finite element simulation analysis can be used to judge the feasibility of the experiment. The fabrication and experimentation of the piezoelectric composite are conducted through simulation analysis. The traditional and the modified 1-3 type piezoelectric composites are fabricated by using the dice-filled method. The length, width, and thickness of each material sample are 63, 29.5, and 3 mm, respectively. The width of each piezoelectric column is fixed to 1 mm. The specific preparation process of modified 1-3 is shown in Figure 4.

1. Cut the sample size. PZT-5A piezoelectric ceramic block (105 mm × 105 mm × 7.5 mm) is cut into a sample with dimensions of 63 mm × 29.5 mm × 3 mm. The ceramic sheet is cut in accordance with the sample size. The cutting blade cuts laterally along the wide side of the ceramic while retaining the ceramic substrate.
2. Apply 618 epoxy resin. The cut samples are washed ultrasonically. After wiping and drying, 618 epoxy resin is perfused and cured at room temperature in a dry place for 24 h.
3. The second cutting of piezoelectric composite. The surface of the epoxy pouring material is polished and smoothed. The material is placed into the cutting machine again for the second cutting, and the blade cuts longitudinally along the long side of the composite.
4. Pour the 704 flexible silicone rubber. The cut ceramics are cleaned through ultrasonic wiping and drying and then poured into the 704 silicone rubber.
5. Use cutting blades to grind away excess composite substrates.
6. Coat silver electrode. The composite sample is polished and leveled. The upper and lower surfaces of the material are coated with conductive silver slurry so as to prepare the modified 1-3 piezoelectric composite sample.

**Figure 5:**
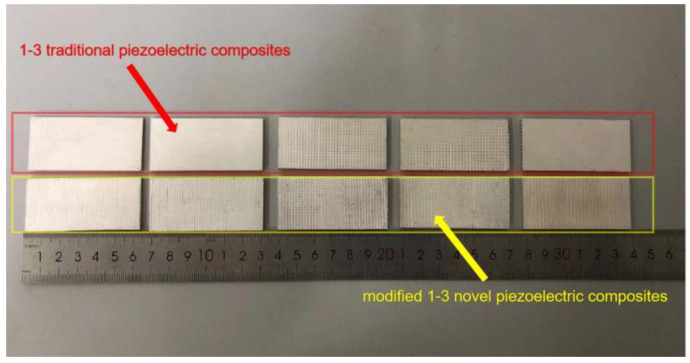
Sample images of two kinds of piezoelectric composite.

For data comparison, the traditional 1-3 type piezoelectric composites are prepared using dice-filled technology [17,28]. The two typ es of samples prepared are shown in Figure 5. The red frame represents the traditional 1-3 type piezoelectric composite, and the yellow frame represents the modified composite. From left to right, the polymer widths of the two samples are 0.2, 0.4, 0.6, 0.8, and 1.0 mm.

## 3. Results and Discussion

### 3.1. Finite Element Simulation Results of Materials

#### 3.1.1. Properties of 1-3 Type and Modified 1-3 Type Composites

**Figure 6:**
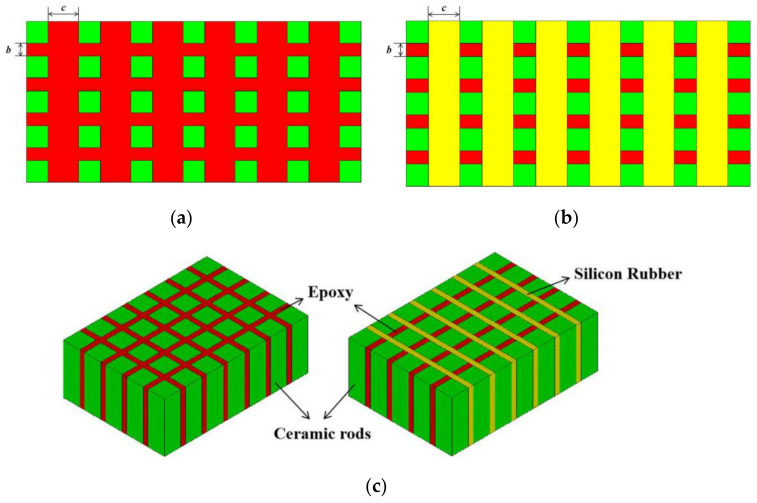
Polymer width settings of traditional 1-3 type (**a**), novel 1-3 modified type (**b**), and their 3-D FEA models (**c**).

**Figure 7:**
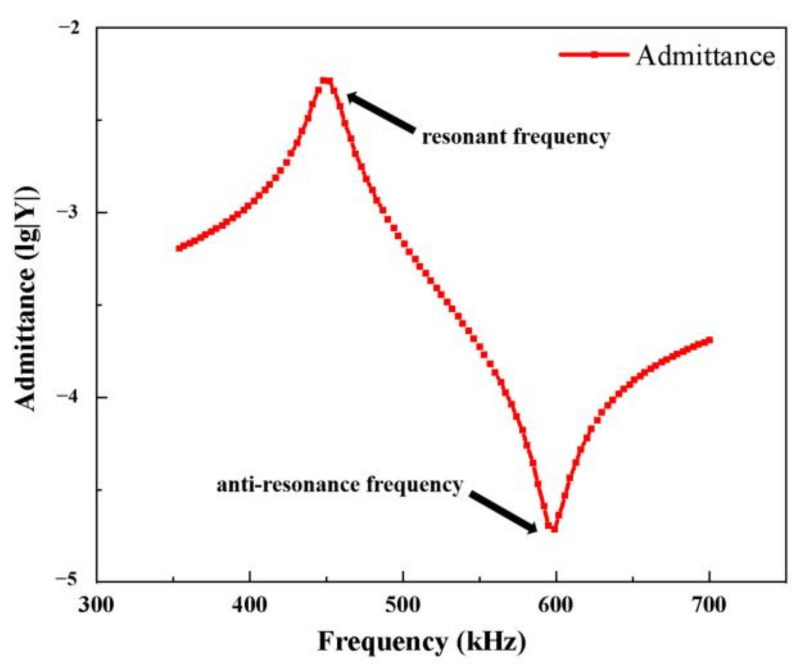
Admittance curve of modified 1-3 piezoelectric composite (*c* = 0.6 mm).

In Figure 6, the widths of the piezoelectric column in the modified 1-3 piezoelectric composite material, epoxy resin, and silicone rubber are set to 1 mm, *b*, and *c*, respectively. The polymer phase widths of *b* and *c* are changed simultaneously (*b = c*). The volume fractions of the three in the composite are obtained (Table 2). The admittance curve of piezoelectric composite is obtained through finite element simulation (Figure 7). Figure 7 shows the calculation results when the polymer width of the modified 1-3 material is 0.6 mm. The resonant frequency of the piezoelectric oscillator corresponding to the admissibility peak point is 448 kHz, and the anti-resonance frequency corresponding to the lowest point is 598 kHz.

**Figure 8:**
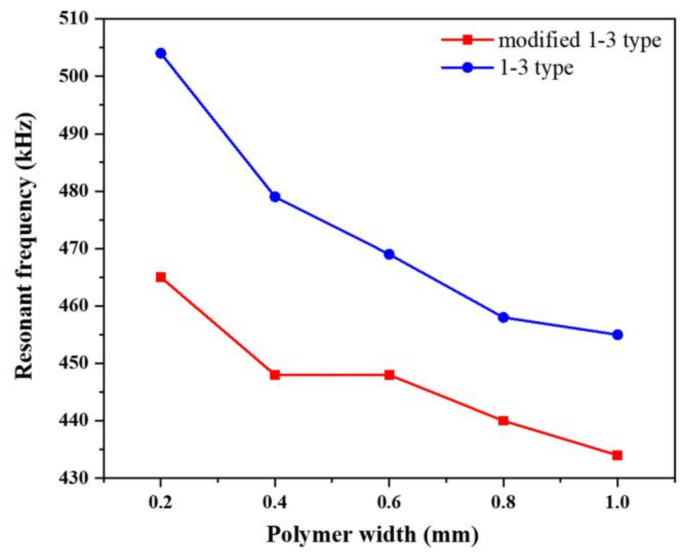
Effect curve of polymer width on resonant frequency of two types of composites.

The resonant frequency is the frequency that affects the maximum response of the piezoelectric transducer due to external excitation. In accordance with the different widths of the polymer phase, the resonance frequencies of the two types of composite are anisotropic (Figure 8) and gradually increase with the decrease in the polymer phase volume fraction and the resonance frequency of the two composites. For the traditional 1-3 type composite, the change of polymer phase volume fraction on the resonance frequency of the vibrator is within 0.2–1.0 mm, with the resonance frequency maximum change being approximately 50 kHz. By contrast, the change of the modified type 1-3 composites is only 30 kHz, with a relatively flat oscillator variation rate compared with 1-3 traditional piezoelectric composites. The low resonant frequency allows the modified piezoelectric composites to reach the resonance point easily.

The electromechanical coupling coefficient of thickness, which is usually expressed by *k*_t_, characterizes the degree of mutual coupling between mechanical energy and electrical energy when piezoelectric materials undergo stretching vibration in the thickness direction. As a transducing material, we assume *k*_t_ to be an extremely high value to improve the energy conversion of the material. *k*_t_ can be expressed by Equation (1) [30]. In Equation (1), *f*_s_ is the resonance frequency and *f*_p_ is the anti-resonance frequency.

(1) $$kt=\sqrt{\frac{fp^{2}-fs^{2}}{fp^{2}}}$$

For traditional 1-3 piezoelectric composites, piezoelectric ceramic phase and epoxy resin phase are mostly used as the material composition structure. The width of the polymer phase changes the electromechanical coupling coefficient of the material in a different manner. Therefore, exploring the effect of the polymer spacing on the *k*_t_ value of the two composites is necessary. The *k*_t_ value of the two types of composites with different polymer widths can be obtained using Equation (1) by changing the polymer widths of the two piezoelectric composites while keeping the other parameters of the composites unchanged.

**Figure 9:**
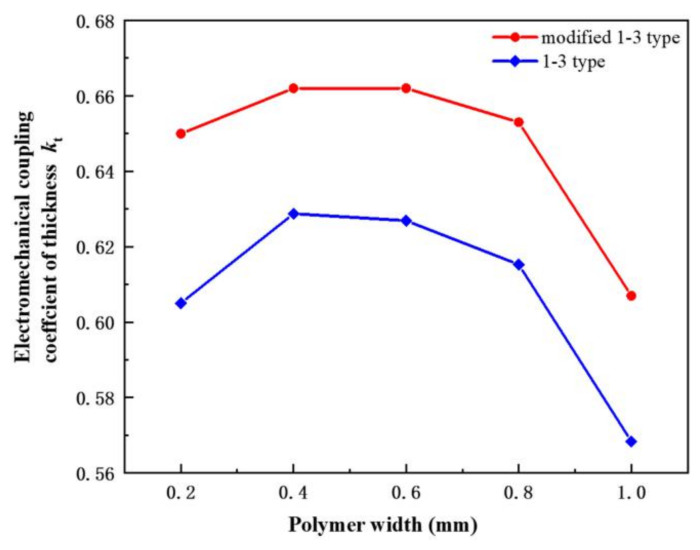
Comparison curve of electromechanical coupling coefficient of thickness with different polymer width.

As shown in Figure 9, the two composites rise in the polymer width range of 0.2–0.4 mm, and the electromechanical coupling coefficient peaks for the 1-3 type composites. In the range of 0.4–0.8, the electromechanical coupling coefficient of modified 1-3 materials decreases gently. When the gap width of the polymer phase is larger than 0.8, the electromechanical conversion ability of the two composites decreases rapidly. Therefore, the volume fractions of the ceramic phase and polymer phase are crucial to limit the electromechanical coupling coefficient of the thickness of the composites. The width of the modified 1-3 polymer composite is between 0.4 and 0.6 mm, the change of electromechanical coupling coefficient is unremarkable, and the traditional 1-3 material curve starts to decrease through the FEA. Thus, we hypothesize that flexible polymers can achieve decoupling effect, which allows the material to reduce energy loss in vibration.

From the curve of the overall trend, the electromechanical coupling coefficient of modified 1-3 piezoelectric composites is obviously higher than that of the traditional one, proving the former’s superior mechanical and electrical conversion properties. Adding the flexible phase after the modification of new materials is expected to significantly improve the launch of a new type of transducer and receiving sensitivity.

**Figure 10:**
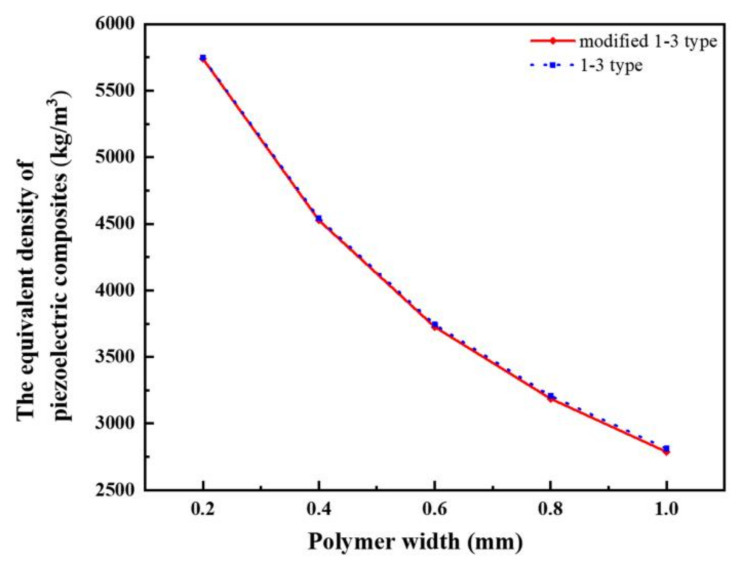
The change curve of equivalent density of the two types of composite.

In accordance with Table 1, we can calculate the equivalent density of each composite with different polymer widths. The equivalent density *ρ* is obtained by weighting the proportion of each constituent phase and its volume, which can be expressed as Equation (2). The density curves of the two materials are obtained, as shown in Figure 10.

*ρ = ρ*_c_*V*_c_ + *ρ*_e_*V*_e_ + *ρ*_s_*V*_s_ (2)

where *ρ* is the density of modified 1-3 type piezoelectric composite; *ρ*_c_, *ρ*_e_, and *ρ*_s_ are the densities for piezoelectric phase, 618 epoxy resin, and 704 silicone rubber, respectively; and *V*_c_, *V*_e_, and *V*_s_ are the volume fractions of piezoelectric phase, epoxy resin, and silicone rubber, respectively.

Figure 10 depicts the relationship between the equivalent densities of the two piezoelectric composites and the width of the polymer phase. The densities along the index form drop with the gradual increase in width. When the polymer width exceeds 0.4 mm, the steepness of decline curve tends to be smooth. The two curves mostly overlap due to the densities of 618 epoxy resin and 704 silicone rubber. From the perspective of data, the 1-3 piezoelectric composites have greater equivalent density than the modified one. The rigidity of the piezoelectric composite is appropriately reduced.

Characteristic impedance is an acoustical physical quantity introduced in lumped parameter analysis of circuit systems. For acoustic transducers, the closer the acoustic impedance of the material is to water (approximately 1.5 MRayl), the better the acoustic match between the system and water when the system works, thereby helping to reduce system errors. Combined with Table 1 and Equation (2), the characteristic impedance *Z* of the two composite materials can be obtained by using Equation (3):

(3) $$Z=2f_{p}t\rho$$

where *f_p_* is the anti-resonance frequency of the material, *ρ* is the equivalent density of the material, and *t* is the thickness of piezoelectric composite.

**Figure 11:**
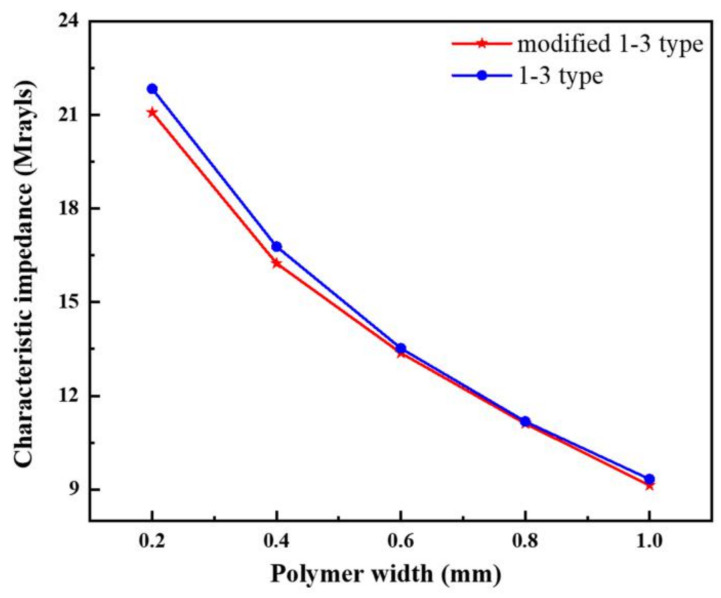
Characteristic impedance curve of two types of composites.

Figure 11 depicts the difference in the characteristic impedance variations of the two composites. In the composites, the volume fraction of the polymer phase increases, and the acoustic impedance decreases with the increase in the polymer width. The modified 1-3 piezoelectric composite material has a low impedance due to the low intrinsic impedance of silicone rubber. This condition makes it easier to match with water acoustically compared with the conventional one.

**Figure 12:**
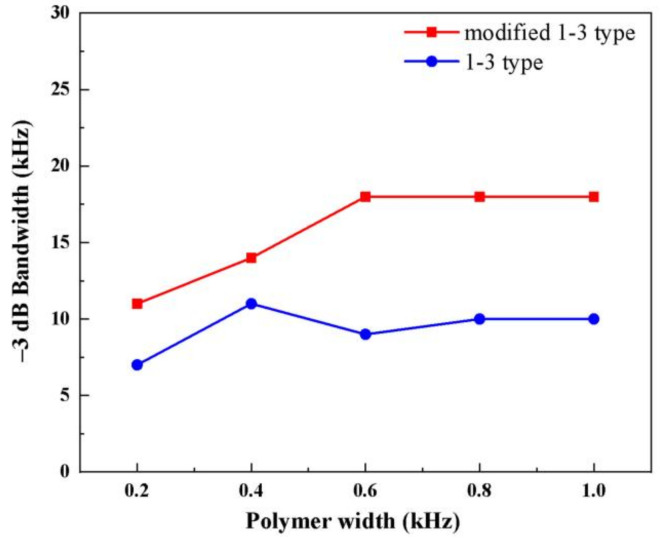
−3 dB bandwidth curves of the two types of composites.

Figure 12 shows the −3 dB bandwidth curves of the two composites at different polymer widths. The modified composite adds flexible polymer phase, which promotes the mechanical vibration on the left and right sides of each piezoelectric rod. Thus, the modified composite has a larger mechanical loss, reduced mechanical quality, and improved bandwidth compared with the traditional 1-3 type.

#### 3.1.2. Decoupling Characteristics of Modified 1-3 Type Composite

**Figure 13:**
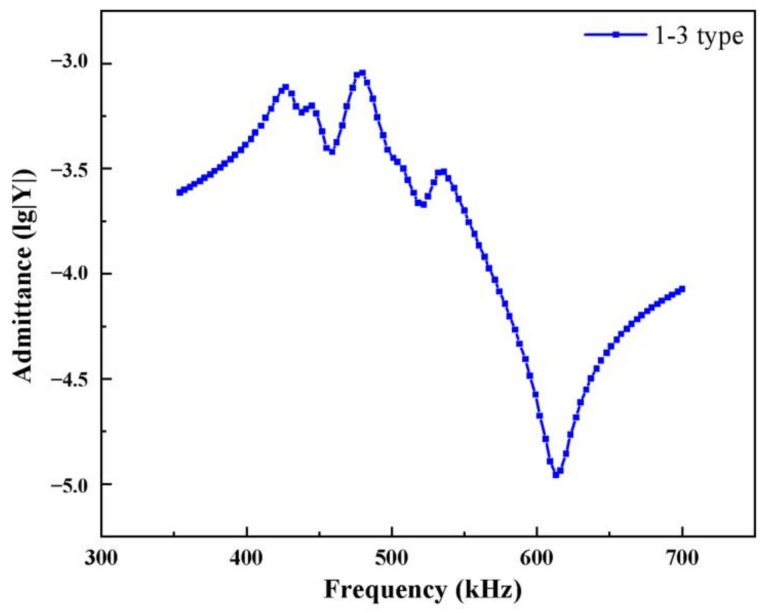
Admittance curves of 1-3 type when coupling harmonics are generated.

Traditional two-phase type 1-3 piezoelectric composites are usually made of PZT ceramics and rigid polymers. When a voltage signal is applied to the upper and lower surfaces of the composite, the piezoelectric composite is affected by the lengthwise direction of the tensile vibration inside each pillar due to the inverse piezoelectricity of the piezoelectric material. The piezoelectric rods drive the nearby rigid polymer to produce coupled vibrations (Figure 2). When the width of the polymer joints is not uniform, the vibration mode of 1-3 piezoelectric composite will no longer be the simple vibration mode of thickness *d*_33_. In accordance with the simulation results in the previous section, the polymer width of the composites can achieve a good thickness electromechanical coupling coefficient within the range of 0.4–0.6. Thus, we set the longitudinal width *b* to 0.6 mm and changed the width of the transverse polymer *c*, as shown in Figure 6a (set width *b* = 0.6 mm, *c* = 1.4 mm), with constant piezoelectric column width and *b* not equal to *c*. The final admittance curve is shown as coupled harmonics producing nonthickness vibrations, as shown in Figure 13. This phenomenon makes it necessary to consider the consistency of the polymer width when designing traditional 1-3 piezoelectric composite in most cases.

As shown in Figure 13, the vibration factor in the 1-3 type piezoelectric composite is composed of the piezoelectric phase and the polymer phase. When the polymer width is small, the piezoelectric column is mechanically clamped, and the overall energy conversion performance of the composite is enhanced. When the polymer is wide, each piezoelectric rod drives the polymer to produce coupling vibration through longitudinal expansion, and the energy conversion of the thickness vibration causes a loss.

**Figure 14:**
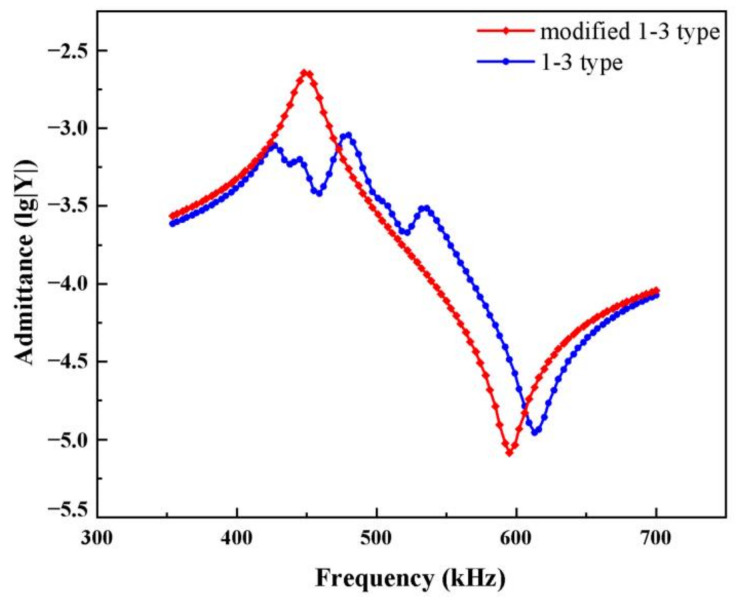
Decoupling effect of modified 1-3 piezoelectric composite.

Similarly, the modified 1-3 piezoelectric composites under the same conditions are shown in Figure 6b (*b* ≠ *c*). A new type piezoelectric composite is added to the structural design of the flexible polymer silicone rubber. The decoupling effect is evident in the composites because the Young’s modulus of the silicone rubber is smaller than that of the rigid polymer epoxy resin. The finite element simulation of the admittance curve is shown in Figure 14 (set width *b* = 0.6 mm, *c* = 1.4 mm), where the vibration modes can be concentrated to the thickness of the single direction, and the generation of coupling clutter is greatly reduced.

In Figure 14, the coupling difference between the two composites are depicted. The modified one has obvious decoupling function. We assume that the modified composites have many concentrated vibration modes under different horizontal and vertical polymer widths while maintaining certain material quality.

The electromechanical coupling coefficient of thickness and maximum admittance modulus of modified 1-3 type composite are investigated when the width of silicon rubber changes. This process is performed to explore the effect of increase in flexible polymer width on the properties of this composite. The longitudinal polymer width *b* is constant, and the lateral polymer width *c* is taken as the variable factor (Table 3).

**Figure 15:**
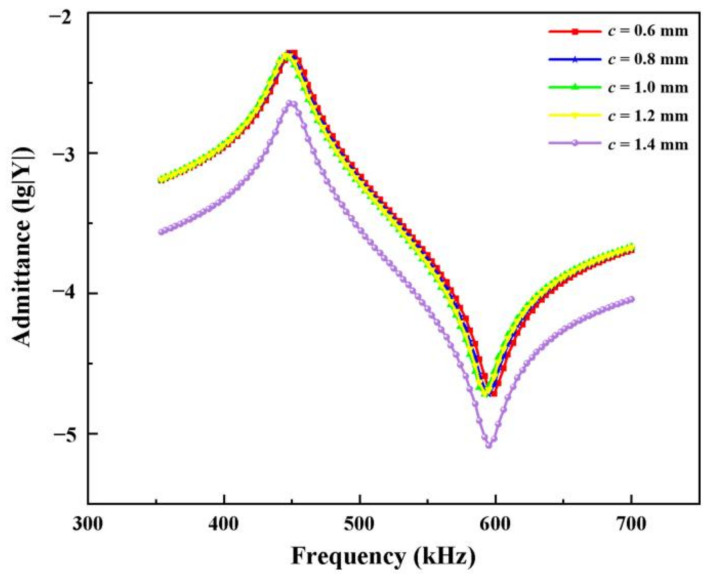
Admittance curves of modified 1-3 type composites with different silicone rubber widths.

Figure 15 depicts the admittance curves of modified 1-3 type composites with different transverse flexible polymer widths. With the increase in the width of the lateral silicone rubber, the volume fraction of the proportion in the material gradually increases. However, several types of composite material admittance curves do not appear in the redundant coupling resonance. This condition indicates that the flexible polymer phase can achieve the decoupling effect and maintain the pure vibration mode of the composite. In Table 3, different horizontal widths of the modified polymer 1-3 type piezoelectric composite bring similar electromechanical coupling coefficients, and the basic remains at around 0.660. This condition shows that this composite has similar electromechanical conversion ability with different lateral and vertical polymer widths.

**Figure 16:**
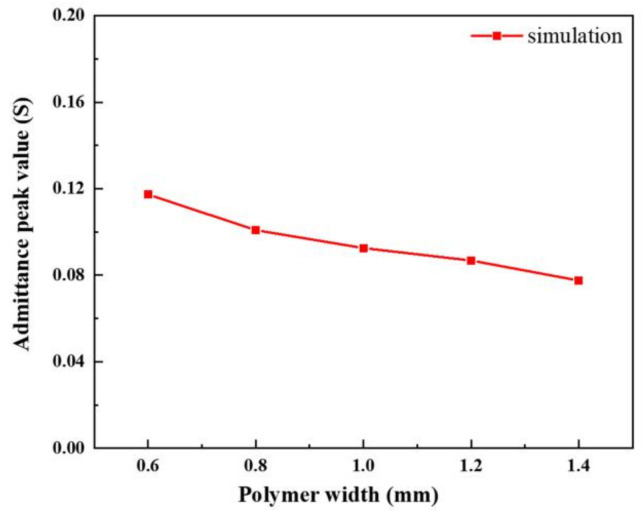
Relationship between transverse polymer width and peak admittance.

The peak value of admittance modulus is the maximum current generated in the piezoelectric composite when the system has the minimum impedance. This value can be used to describe the difficulty of current transmission in the system when the piezoelectric composite vibrates at the resonance point. Figure 16 shows the relationship curve between the width of the transverse polymer and the peak admittance of the modified 1-3 piezoelectric composites. When the overall dimensions of the material are constant, the more silicon rubber is added, the smaller the volume fraction of the ceramic phase, and the more obvious the blocking effect of the piezoelectric composites’ current, resulting in the decrease in conductivity. Therefore, flexible polymers, such as silicon rubber, have good decoupling effect and allow the material to maintain in high purity vibration mode. However, practical application scenarios make it necessary to consider their conductivity.

The FEA method shows that the modified 1-3 piezoelectric composite material has greater practicability than the traditional 1-3 PZT- epoxy piezoelectric composite. The introduction of flexible polymer silicone rubber phase increases the electromechanical coupling coefficient of the thickness and strengthens the electromechanical conversion capacity of the composite, thereby improving the transmission and reception performance of the transducer. Low characteristic impedance enables the material to better match with the water acoustics so as to transmit or receive underwater acoustic waves. The flexible polymer promotes the mechanical loss of each piezoelectric rod and enhances the composite’s bandwidth. The vibration mode impurity of the traditional two-phase 1-3 piezoelectric composite material is obviously improved. Silicone rubber polymer with low Young’s modulus is used to remove the clutter generated by coupling vibration, and the vibration mode is restored to a single pure thickness. The modified composite is valuable in the preparation of the sensitive element of the new transducer.

### 3.2. Experimental Test Results of the Materials

Two types of composites are prepared and tested experimentally with an impedance analyzer (Agilent 4294A, Agilent Technologies, Inc., Santa Clara, CA, USA). The admittance curve obtained through the experiment shows the resonance frequency of the composite, where the actual thickness electromechanical coupling coefficient can be analyzed. The characteristic impedance can be calculated on the basis of the actual antiresonance frequency and the equivalent density in the previous section. The −3 dB bandwidth can be directly read in the impedance analyzer. The detailed data are shown in Table 4.

**Figure 17:**
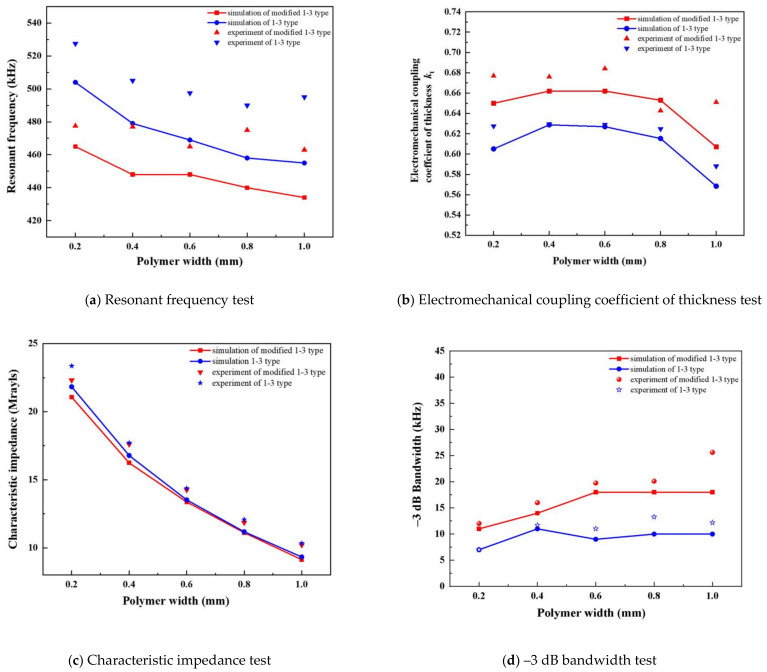
Experimental tests of composites with different polymer widths: (**a**) Resonant frequency test, (**b**) Electromechanical coupling coefficient of thickness test, (**c**) Characteristic impedance test, (**d**) −3 dB bandwidth test.

The actual measured data are plotted into the finite element simulation diagram for comparison (Figure 17). The experimental results of the four groups are consistent with the trend of simulation. The modified 1-3 piezoelectric composite has obvious advantages in simulation and experiment.

In Figure 17a, the measured composites and modified ones do not completely coincide with the simulation curve. The presumed reason is that the material parameters set in the simulation are slightly different from those selected in the experimental tests. With the gradual increase in polymer width, the resonant frequency of the modified 1-3 piezoelectric composites shows a slight increase trend when the polymer width is 0.8 mm, but the increased value remains lower than that of the traditional composites. On the whole, the impedance analyzer test verifies that the modified 1-3 piezoelectric composite is easier to reach the resonant point than the traditional one, and the working frequency is relatively low. However, in the transverse comparison, extremely wide or narrow polymer settings increases the resonance frequency of the composite. The polymer width set at approximately 0.6 mm can reduce the resonance frequency to its lowest.

Figure 17b describes the measurement of the actual electromechanical coupling coefficient of the thickness of the two types of composites. The addition of flexible polymer phase promotes the mechanical vibration of each ceramic column in the modified 1-3 piezoelectric composites. The simulation curve is consistent with the measured data. The polymer width is 0.6 mm, and the thickness of the electromechanical coupling coefficient is 0.684. Compared with the same condition of 1-3 type piezoelectric composite, the electromechanical coupling coefficient of the modified piezoelectric composite can be promoted by approximately 8.0%. The electromechanical coupling coefficients of the thickness of the two composites decrease with the increase in polymer width. However, the modified composite remains stable at approximately 0.65, and the value for the traditional one decreases obviously with the lowest value being 0.588.

Figure 17c shows the test data of the characteristic impedance of the two materials. The measured data are consistent with the simulation analysis. Compared with the 1-3 type piezoelectric composite, the characteristic impedance of the new composite can decrease by up to 4.5%. The larger the polymer width, the lower the difference of the characteristic impedance between the two types of composites.

Figure 17d shows the measurement results of −3 dB bandwidth of the two types of composites. On the whole, the bandwidth of the modified composites is always higher than that of the traditional one. When the set width is large, the advantage of the −3 dB bandwidth of modified composites is obvious. When the polymer width exceeds 0.8, the difference between the simulation and experiment gradually increases. When the polymer width is 1.0 mm, the −3 dB bandwidth of the modified composite material can be as high as 25 kHz, which varies greatly with the simulation results. The epoxy resin mainly suppresses the plane vibration mode and performs mechanical clamping. However, an extremely large set value of its width results in the coupled vibration of piezoelectric phase and epoxy phase. This condition causes complex vibration modes of the modified 1-3 piezoelectric composites, ultimately resulting in an unexpected increase in bandwidth at that point.

**Figure 18:**
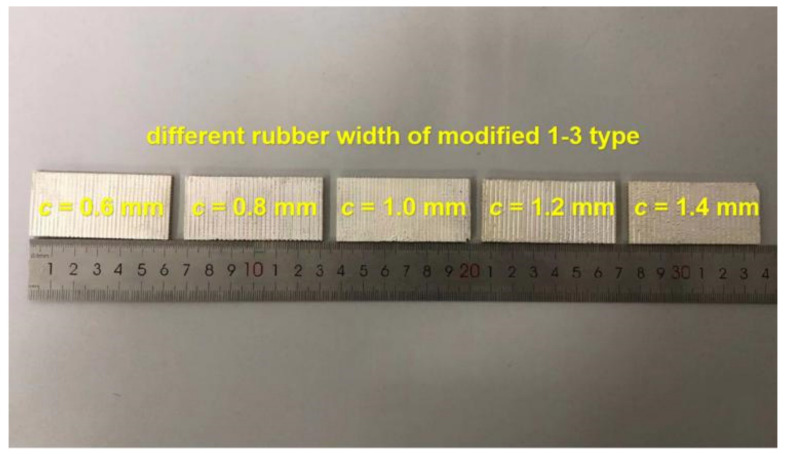
Sample diagram of modified 1-3 piezoelectric composites with different silicon rubber widths.

**Figure 19:**
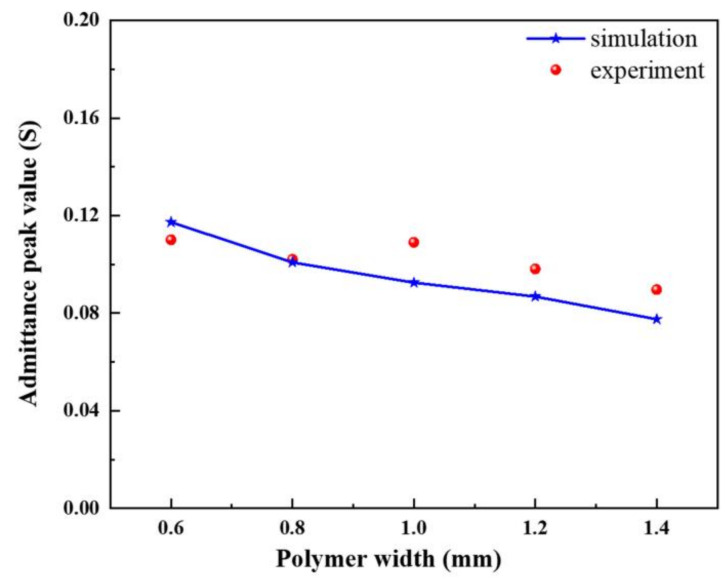
Test of admittance peak at different silicone rubber widths.

In accordance with the finite element simulation conclusion in the previous section, five modified 1-3 piezoelectric composites with different silicon rubber widths are fabricated in the experiment, as shown in Figure 18. Figure 19 shows the comparison between the measured data of the admittance peak value of the modified composite and the simulation data after changing the width of the transverse silicone rubber. The data in the figure show that the two are consistent.

In this work, the simulation and actual test data are slightly different. This condition may be due to three factors [17]. The first error is generated by the cutting instrument. In this experiment, we select PZT-5A with different polymer widths for cutting. The blade thicknesses of the laboratory cutting instrument are 0.20, 0.50, and 0.80 mm. Thus, we need to cut the ceramics with large gaps by changing the blade, and the target width is cut multiple times, which may cause errors in the gap after cutting. The second error is caused by the parameters of the raw materials. During the simulation, the information inputted into the material library is provided by the manufacturer, as shown in Table 1. The actual value we used may not be exactly the same as the value provided by the manufacturer, resulting in slight parameter errors. The third error may be caused by the experimental operation. The material is in an ideal external environment when the simulation is performed on ANSYS, and the inside of the material is extremely uniform for calculation. For actual materials, defects are inevitably caused by small impurity bubbles in the epoxy resin liquid and slight changes in the thickness of the material after polishing. These conditions make the difference between the simulation and experiment and need to be further studied.

In summary, the three-phase modified 1-3 piezoelectric composite is composed of piezoelectric phase, epoxy phase, and rubber phase. We use FEA simulation and experiment to prove the advantages of this composite over the traditional 1-3 piezoelectric two-phase composite. Compared with the traditional one, the modified composite has lower resonant frequency and higher electromechanical coupling coefficient of thickness, indicating that this novel composite has a higher electromechanical conversion ability. The silicone rubber enhances the bandwidth by increasing the mechanical loss of the piezoelectric composite, thereby achieving the decoupling effect. Thus, it helps to expand the application range of 1-3 piezoelectric composite.

## 4. Conclusions

In this paper, the modified 1-3 type of three-phase piezoelectric composite material is fabricated using the “dice-filled” method. FEA is conducted to compare the properties of this composite and the traditional PZT-epoxy 1-3 type piezoelectric. The modified composite has low resonance frequency, acoustic impedance, high bandwidth, and electromechanical coupling coefficient of thickness. The flexible polymer phase can achieve decoupling effect simultaneously, and its advantages are verified through experiments. For the modified material, the electromechanical coupling coefficient of the thickness is up to 0.684, which can be improved by 8.0% compared with the traditional 1-3 type composites. The characteristic impedance can be reduced by 4.5%. The modified composites with a moderate width of silicone rubber can maintain relatively smooth admittance curve with concentrated vibration modes of thickness and better decoupling effect. In conclusion, the modified 1-3 piezoelectric composite is advantageous and practical, thereby providing a solid foundation for the preparation of new piezoelectric transducers in the future.

## Figures and Tables

**Table 1:** Parameters of each component phase of composite.

Parameter	PZT-5A	Epoxy	Silicon Rubber
*ρ* (kg/m^3^)	7750	1050	1000
*c*^E^_11_(10^10^ N/m^2^)	12.1	0.36	0.004
*c*^E^_12_(10^10^ N/m^2^)	7.54	0.138	0.0023
*c*^E^_13_(10^10^ N/m^2^)	7.52	278	4 × 10^5^
*c*^E^_33_(10^10^ N/m^2^)	11.1	−97	2.3 × 10^5^
*s*^E^_11_(10^−12^ m^2^/N)	16.4	278	4 × 10^5^
*s*^E^_12_(10^−12^ m^2^/N)	−5.74	−97	2.3 × 10^5^
*s*^E^_13_(10^−12^ m^2^/N)	−7.22	/	/
*s*^E^_33_(10^−12^ m^2^/N)	18.8	/	/
*e*_31_	−5.4	/	/
*e*_33_	15.8	/	/
*ε*^S^_33_/*ε*_0_	/	4	3.3
*ε*^T^_33_/*ε*_0_	/	4	3.3

**Table 2:** Volume fraction table of corresponding component phases of polymer phase width.

Polymer Width (mm)	Ceramic Volume	Epoxy Volume	Rubber Volume
0.2	0.701	0.135	0.164
0.4	0.521	0.198	0.281
0.6	0.402	0.229	0.369
0.8	0.322	0.242	0.437
1.0	0.263	0.245	0.492

**Table 3:** The effect of transverse polymer width on material properties.

Rubber Width Properties	Resonant Frequency *f*_s_ (kHz)	Electromechanical Coupling Coefficient of Thickness *k*_t_	Maximum Admittance Modulus (S)
0.6	448	0.662	0.117
0.8	448	0.658	0.101
1.0	444	0.660	0.093
1.2	444	0.660	0.087
1.4	444	0.660	0.078

**Table 4:** Performance test data of 1-3 type and modified 1-3 type piezoelectric composites.

Materials Type	Polymer Width (mm)	Resonant Frequency (kHz)	Anti-Resonance Frequency (kHz)	Electromechanical Coupling Coefficient of Thickness	Characteristic Impedance (Mrayls)	−3 dB Bandwidth (kHz)
1-3 type	0.2	527.5	677.5	0.628	23.36	7.01
	0.4	505	650	0.630	17.71	11.70
	0.6	497.5	640	0.629	14.37	11.03
	0.8	490	627.5	0.635	12.08	13.28
	1.0	495	612	0.588	10.24	10.19
Modified 1-3 type	0.2	477.5	648	0.677	22.31	12.03
	0.4	477	648	0.676	17.60	16.01
	0.6	465	637.5	0.684	14.25	19.76
	0.8	475	620	0.625	11.85	20.12
	1.0	463	610	0.649	10.20	25.62

## Data Availability

The data presented in this study are available on request from the corresponding author.
